# Liver complications in celiac disease

**Published:** 2011-05-01

**Authors:** Mohammad Reza Zali, Mohammad Rostami Nejad, Kamran Rostami, Seyed Moayed Alavian

**Affiliations:** 1 Research Center for Gastroenterology and Liver Disease, Shahid Beheshti University of Medical Sciences, Tehran, IR Iran; 2 School of Medicine, University of Birmingham, Birmingham, UK; 3 Baqiyatallah Research Center for Gastroenterology and Liver Disease, Tehran, IR Iran

**Keywords:** Celiac disease, Liver, Epidemiology

## Abstract

Celiac disease (CD) is characterized by sensitivity to gluten, which is found in dietary wheat, barley, and rye. Many extra-intestinal manifestations have been described in association with CD. Liver disease and CD share widespread risk factors. Liver disorders such as autoimmune hepatitis, elevation of liver enzyme levels, primary biliary cirrhosis, nonspecific hepatitis, primary sclerosing cholangitis, and nonalcoholic fatty liver disease have been reported in patients with CD. In this review, we provide information regarding liver disorders that may be found in association with celiac disease and the effect of the treatment of CD on these disorders.

## Background

Celiac disease (CD) is defined as a condition that affects the morphology of the mucosa of the small intestines, and it is improved if the patient consumes a gluten-free diet and relapses if gluten is reintroduced in the diet [1]. The prevalence of CD is high in the general Iranian population (1 in 166) [2], and the disease is currently considered the result of a complex interplay between inherent and environmental factors. The typical or classical form of CD is due to the interaction between gliadin and antibodies to tissue transglutaminase (tTG), and it results in the flattening of the villi in the small intestinal mucosa [3]. Although CD is known to affect the small intestine, it is a multisystem disorder and can involve other organs such as the skin, tfhyroid, pancreas, heart, liver, joints, muscles, bones, the reproductive system, the central and peripheral nervous systems [4][5][6][7][8][9][10][11][12][13].

The occurrence of liver impairment in CD is well established and must be regarded as one of the various extraintestinal presentations of gluten-sensitive enteropathy [14][15][16][17]. The association between CD and liver manifestations was first reported in 1977 [14]. In this study, 30 of 74 adults newly diagnosed with CD had elevated levels of serum aminotransferase enzymes, which normalized after adherence to a gluten-free diet in most cases. In this study, signs of reactive hepatitis were noted in 5 of 13 patients, and different types of histologic lesions were found in 7 patients. Since the 1990s, a close association between CD and autoimmune liver disease has been clearly indicated in relevant studies [18][19][20][21].

Searches were performed in PubMed and SID (for Persian papers) for articles published in English- and Persian-language journals from 1977 to November 2010; the following keywords were used alone or in combination: "celiac disease," "liver disorders," "liver abnormality," "liver injury," "hepatitis," "anti-tTG," "anti-endomysial," and "cholangitis." The aim of this review is to discuss the major forms of liver abnormalities associated with CD and to evaluate the prognosis of these abnormalities.

### Liver Dysfunction related to CD

Patients with CD have damaged gut mucosa, which can lead to malabsorption and increased permeability. A wide variety of liver injuries may occur in CD [22], and the principal conditions are listed in Table 1.

**Table 1:** Characterization of cryptogenic liver disorders related to CD

**Cryptogenic liver disorders**	High liver enzymes due to gluten induced reactive hepatitis
**Autoimmune liver disease**	Autoimmune hepatitis (AIH)
	Autoimmune overlap syndrome
	Primary sclerosing cholangitis (PSC)
	Primary biliary cirrhosis (PBC)
	Nonalcoholic fatty liver disease (NAFLD)
	Nonalcoholic steatohepatitis (NASH)
	Hepatitis C virus (HCV)-related liver disease

Conditions such as nonalcoholic fatty liver disease (NAFLD), nonalcoholic steatohepatitis (NASH), hepatitis C virus (HCV)-related liver disease, and hepatitis B virus (HBV)-related liver disease are very common in the general Iranian population [23][24][25], and their incidence in patients with CD is likely a coincidence rather than a true correlation. Recently, 2 different types of liver injury, namely cryptogenic liver disorder (mild or severe type) and autoimmune liver disorder, have been found to be strongly related to CD. Cryptogenic liver disorder can be distinguished from autoimmune liver disorder on the basis of its positive response to gluten-free diet (GFD).

### Cryptogenic liver disorders 

1. Mild liver damage (gluten-induced hepatitis)

The first report of gluten-induced hepatitis, published in The Journal of Pediatric Gastroenterology and Nutrition in 1986, was the case of a young girl with persistent cryptogenic elevation of serum aminotransferase levels and mild inflammation of the portal tract [26]. A diagnosis of CD, suggested in this case by a high titer of anti-reticulin antibody, was confirmed by duodenal biopsy. Bardella et al. performed a similar study and found that 13 (9%) of 140 screened patients tested positive for antigliadin antibody (AGA) and anti-endomysial antibody (EMA). The relative risk of CD in these patients (18.6%) was significantly greater than that in the general population. Antibodies associated with CD disappeared after 12 months of consuming a GFD, but liver enzyme levels normalized in only 1 patient [27]. This form of CD was once called "gluten-induced hepatitis" [28] and is now suggested to be celiac hepatitis, which is characterized by mild periportal inflammation with Kupffer cell hyperplasia, mononuclear cell infiltration, absence of any clinical features suggesting chronic liver disease, absence of hypergammaglobulinemia, absence of serum autoantibodies, presence of mild lobular and portal tract inflammation, and absence of hepatomegaly, splenomegaly, or both; this form is reversible if a GFD is consumed. In the majority of patients with hypertransaminasemia at diagnosis, liver enzymes returned to normal levels within 6 months of starting a strict GFD [29] and in almost all cases, returned to normal levels within 12 months of gluten withdrawal. Occasionally, hypertransaminasemia might be the only sign of CD, which is manifested without any gastrointestinal (GI) symptoms. The results of a study by Volta et al. showed that 9% of patients with increased levels of transaminase of unknown origin had asymptomatic CD [28]. Furthermore, evaluation of 110 patients with cryptogenic hypertransaminasemia showed that 10% of patients with elevated transaminase levels were also positive for silent CD [29].

On the basis of the high prevalence of CD (9.3%) in patients with unexplained increases in serum transaminase levels, Bardella, et al. confirmed that cryptogenic hypertransaminasemia could be a possible extraintestinal sign of CD [30]. Furthermore, investigators found that children with unknown causes of hypertransaminasemia had asymptomatic CD. By consuming a GFD, patients experienced rapid improvements in both hepatic and intestinal biochemical/histologic signs, thus confirming that the liver damage was gluten-dependent [31]. In a few studies, it was reported that some patients on a GFD for 12 months newly experienced increases in their liver enzyme levels, and this increase was probably because of the high quantities of lipids contained in some gluten-free foods [31][32].

2. Severe liver damage

Severe histological diseases, including chronic hepatitis, severe fibrosis, and cirrhosis have been reported in adults and children [14][25][31]. CD was detected in some patients with severe liver damage of unknown origin, and surprisingly, clinical improvement in the liver condition was noted when the patients consumed a GFD [33][34][35]. The prevalence of CD in patients with chronic liver disease is higher than in the general population. Lindgren et al. reported that in 327 patients with chronic liver disease, the prevalence of CD was 1.5%, which is 15 times higher than that in the general population [36]. In a Finnish study, CD was reported in 4 adult patients (3 men and 1 woman) with severe liver disease, who were waiting for liver transplantation [33]. Two of them had progressive hepatitis, congenital liver fibrosis, and massive hepatitis steatosis. Clinical symptoms, including ascites and jaundice, improved in all patients after 6 months of consuming a GFD. Of 185 Finnish patients who underwent transplantation, 8 (4.3%) were found to have CD; this rate is 4-10 times higher than that found in the general population [33]. During the study for a transplant programin a 28-year-old woman with severe cryptogenic liver failure had a complete recovery of liver function after a few months of following a GFD [34].

3. Autoimmune liver diseases

3-1) Primary biliary cirrhosis

The relationship between CD and primary biliary cirrhosis (PBC) is well documented, and it was first reported by Logan, et al. in 1978 [37]. Consequently, PBC patients have been extensively screened for CD. The reported prevalence of CD in PBC varies between 0 and 11%, and about 6% of individuals with CD may be affected by PBC [20][38][39]. In a 12-year epidemiological study of a British population of 250,000, Kingham and Parker [38] accessed a large registry of patients with PBC and CD. They found that CD was identified in 4 of 67 (6%) patients with PBC and that 4 of 143 (3%) patients with CD were affected by PBC. In another study conducted in UK, in which 4,732 CD patients were matched with 23,620 controls, the prevalence of PBC was found to be 0.17% in patients with CD versus 0.05% in controls [39]. This trend was confirmed by 2 large population-based studies from Danish and Swedish cohorts [40]. In these studies, EMA antibody tests were positive in 11% of PBC patients. The titer of mitochondrial antibodies, which are markers of PBC, did not change after gluten removal. Various case reports on the association between CD and PBC have shown that a GFD induces a stable clinical and biochemical improvement because of the normalization of intestinal absorption, but it does not seem to adjust the course of the liver disorder [41][42][43][44][45][46][47][48][49]. However, some studies have shown that there is no association between CD and PBC [50][51], but these studies tended to have small numbers of studied patients. In addition, numerous investigations have reported the positive effect of a GFD in primary biliary cirrhosis, thereby suggesting that all patients with PBC should be screened for CD [8][13][14][15][16][17][18][19][20][52][53][54][55].

3-2) Primary sclerosing cholangitis

No distinguishing autoantibody has been found in patients with primary sclerosing cholangitis (PSC). Using cholangiography as a diagnostic tool, the characteristics of the biliary lesion can be evaluated in biopsy tissue or the appearance of the intra- and extrahepatic biliary tree can be assessed [12]. Several studies have found a positive correlation between PSC and CD; however, these studies involve small numbers of investigated patients, and also, surveillance bias has not been taken into account. This association was first suggested by Hay, et al. in 1988 [56], who described the cases of 3 PSC patients (confirmed by cholangiopancreatography retrograde endoscopy and liver biopsy) with steatorrhea as a severe form of malabsorption. Diarrhea was ameliorated after the diagnosis of CD and consumption of a GFD, but the course of PSC did not improve. In a recent study by Volta, et al. positive anti-EMA antibody was found in 1 of 61 patients with PSC, thus showing a prevalence of 1.6% for CD [20]. The relationship between PSC and CD has not been extensively studied. In a study of 13,818 patients with CD and 66,584 age- and sex-matched individuals from the general Swedish population, the prevalence of PSC in CD patients was 4.46%, a rate 4-8 times higher than that of the general population [57]. In another survey, CD was found in 3% patients with PSC [58].

Subsequently, the association between PSC and CD has been reported in several case reports [59][60][61][62][63]. Liver enzyme levels in a 54-year-old man with CD, PSC, ulcerative colitis, and Hashimoto's thyroiditis were noticeably improved and finally normalized after 14 months of treatment with a GFD (64. A repeated liver biopsy showed marked improvement in liver histological characteristics. In addition, in 2 studies, GFDs were reported to cause a significant improvement in hepatic histological characteristics and cholestasis in 3 patients. The small number of studied cases does not allow the formation of a definite conclusion as to whether diet slows down the progression of this autoimmune liver disorder [57][60]. Further studies are needed to accurately determine the strength of the association between PSC and CD.

3-3) Autoimmune hepatitis

In the late 1970s, sporadic findings of CD in patients with autoimmune hepatitis (AIH) were reported for the first time [65][66][67]. To evaluate the incidence of CD in patients with AIH, Volta, et al. studied the sera of 157 patients with type 1 AIH and 24 patients with type 2 AIH; CD was found in patients with both types of AIH and EMA antibody was identified in 8 AIH patients (4%). CD was diagnosed in 5/8 patients who underwent a duodenal biopsy [19]. However, the benefit of a GFD to the clinical course of AIH patients was not reported. This study was performed through cooperation between the Mayo Clinic (Rochester, USA) and the University of Bologna (Italy).

In another study, the prevalence of CD in patients with AIH was 6.4% [21]. In a multicenter study in Italy, AIH occurred in 1.1% of 909 children with CD and no case was found in either healthy populations or in patients with an alternate gastrointestinal ailment such as Crohn's disease [68]. Jacobsen, et al. studied 101 patients with CD [69] and detected chronic active hepatitis in 5 patients (2.3%) of 37 patients who underwent histologic evaluation. In addition, in a study by Novacek, et al. 3 (1.6%) of 178 patients with CD investigated for the presence of abnormal liver enzyme levels had documented AIH [70]. In a study done at King's College, London, the prevalence of CD in 96 children with AIH was 3.4%, which was significantly higher than expected [71].

4. Autoimmune cholangitis

An association between CD and autoimmune cholangitis (AIC) has been described [72]. Intestinal biopsies of patients with CD and AIC usually show either mild atrophy or an increased number of intraepithelial lymphocytes. In a 60-year-old woman who was evaluated for chronic elevations of serum liver biochemical parameters and unexplained iron deficiency anemia, a diagnosis of CD was made; subsequently, treatment with a GFD led to resolution of these abnormalities [73]. These studies suggest that CD should be considered in all patients diagnosed with AIC, as a GFD may avoid the need for immunosuppressive therapy.

5. Other liver disorders in CD

5-1) Viral Hepatitis

Hepatitis B and C are prevalent in Iran [74][75][76][77]. It is estimated that between 1.2% and 19.7% of the general population have hepatitis B surface antigen (HBs Ag) and 0.12-0.89% have anti-hepatitis C virus antibodies, corresponding to 1.5-2.5 million HBV cases and 0.5 million chronic carriers of HCV [75][76]. In a study recently published in Iran, 88 patients with chronic hepatitis B (CHB) were serologically tested for celiac autoantibodies, and 9 seropositive patients underwent duodenal biopsy [78]. Compared with the general population, the prevalence of celiac autoantibodies in CHB (11.3%) is relatively high, and most autoantibody-positive patients were asymptomatic for CD.

Fifty-five percent of children and 68% of adults do not respond to standard vaccination regimens for hepatitis B virus. This lack of response to hepatitis B vaccine may be related to the genetic background of celiac patients, which seems to be linked to human leukocyte antigen (HLA) DQ2 [79][80]. Some authors have suggested that large follow-up studies with large sample sizes are necessary to clarify how HBV infection affects the development of CD and to identify principal prevention strategies [81][82][83]. There is no convincing evidence that patients with viral hepatitis are at increased risk of developing CD [84][85], but an association between HCV infection and CD in adults has been reported. Indeed, HCV is suggested to be the most common liver disease associated with CD. Two hundred and fifty-nine patients who were consecutively evaluated with chronic hepatitis C (CHC) and 221 healthy volunteers underwent serologic screening for CD, and seropositive patients underwent duodenal biopsy [86]. The results of this study showed that the prevalence of CD in patients with CHC was 1.2%, while only 0.4% of healthy volunteers had CD. In another study, the prevalence of antibodies to tTG in 462 patients with CHC was higher than that in 1,350 healthy controls [87]. A third study showed that the prevalence of CD in 534 patients affected by CHC was 1.3% [88].

A recent study of patients having both CD and HCV described a well-defined route of transmission in most of these subjects, raising the hypothesis that the link between these diseases may be biased by the route of transmission of hepatitis C infection [89]. An association between HCV infection and CD has been hypothesized, but some studies show that there is no correlation between these 2 disorders. Thus, a clear association of CD and HCV is lacking. In a cross-sectional study, 827 multiparous pregnant women were serologically screened for CD and HCV antibodies by enzyme-linked immunosorbent assay (ELISA). Twenty-seven (3.26%) women had antibodies to tTG, but only 2 (0.24%) had antibodies to HCV and one of these also had antibodies to tTG. This result suggests that routine screening of HCV in CD patients is not efficient [90]. Both interferon and ribavirin may enhance type 1 helper T cell immune responses via signal transducers and activators of the transcription-dependent pathway, which subsequently induces the expression of interferon [91][92][93][94]. Cammarota, et al. reported 2 cases of CHC that displayed various features of CD during treatment with interferon. Symptoms and histological disorders improved after the interferon treatment was stopped and a GFD was consumed [92]. Therefore, it is suitable to start treatment for HCV after CD diagnosis, and after a year of GFD, improvement of the intestinal disease can be achieved. Since HCV is a very common disorder and may be correlated to CD, the presence of undetected CD should be ruled out before starting treatment of interferon in combination with ribavirin. Furthermore, CD should be considered in patients with unexplained diarrhea during or after interferon/ribavirin therapy.

5-2) NAFLD/NASH

Some conditions such as NAFLD and/or NASH are very common (up to 25%) in the general population [95], and its occurrence in patients with CD is likely to be a coincidence rather than a true correlation [96]. Some studies declare a clear association between CD and fatty liver and suggest performing serological screening and continually evaluating biochemical abnormalities for CD in NAFLD patients [18][96]. Bardella, et al. investigated 59 patients with hypertransaminasemia and NAFLD, and 38 (64%) were diagnosed with NASH by anti-EMA and anti-tTG antibodies. HLA-DQ typing and endoscopy were performed in 2 anti-EMA positive patients (3.4%) and 6 anti-tTG positive patients (10%). On the basis of histological findings, CD was confirmed only in 2 patients (3.4%) who were positive for both anti-EMA and anti-tTG. After 6 months of a GFD, liver enzyme normalized in both cases [96]. However, other studies do not recognize associations between CD and NAFLD [97]. Nehra, et al. attempted to determine whether any relationship exists between NASH and CD by investigating the serology of 47 NASH patients, in whom NASH was confirmed by liver biopsy. Only one patient was EMA-positive, thus indicating that there is no association between positive CD serology and NASH [97]. In contrast, Valera, et al. found positive CD markers in 3 of 38 NAFLD patients (7.9%), and histological evaluation showed Marsh I in 1 patient. The result of this study has indicated a high prevalence of positive tTG in patients with NAFLD [98]. In an Italian study, the prevalence of silent CD in 59 consecutive patients with NAFLD was 3.4% [99], which suggested that screening with EMA is preferred to tTG antibodies since tTG positivity, in the absence of confirmatory anti-EMA antibodies, is not sufficient to perform diagnostic endoscopy.

5-3) CD and hemochromatosis

CD and hemochromatosis are genetic disorders, but CD is paradoxically associated with iron deficiency anemia. Most published papers regarding these disorders together are case reports [100]. Singhal et al. reported the cases of 2 patients who developed both CD and hereditary hemochromatosis [101]. In the first case, CD was diagnosed first and 8 years later, a routine blood screen showed elevated mean corpuscular and alanine transaminase (ALT) levels. Additional examinations showed transferrin saturation and increased levels of ferritin and iron. Genetic analysis showed homozygosity for the C282Y gene mutation, which confirmed hemochromatosis. Liver biopsy showed an index consistent with hemochromatosis. The patient responded satisfactorily to venesections, with the serum ferritin levels presently maintained around 50 mg/L. The second case was that of a 54-year-old woman referred with abnormal ALT levels, but the rest of the liver profile and other biochemical tests were satisfactory. Screening tests for hepatitis B and C and the autoimmune profile were negative, but her serum ferritin level was noticeably raised. Heterozygosity for the H63D and C282Y mutations was shown in a genetic study. The patient rapidly responded to venesection, but the level of ALT remained high. Because of increasing upper abdominal discomfort, she underwent endoscopy, and histological evaluation confirmed CD. After 3 months of treatment with GFD, her liver disorders resolved completely and her ALT levels normalized [101].

Butterworth et al. investigated 145 CD patients and 187 matched controls for the presence of HFE gene mutations. The number of mutated C282Y and/or H63D genes were significantly higher in patients with CD (48.3%) compared to controls (32.6%). Nevertheless, none of the patients with CD showed a clinical presentation of hemochromatosis. The results of this study showed that HFE gene mutations are more frequent in the CD population and may provide a survival benefit by ameliorating the iron deficiency seen in these patients [102]. The present studies show that there is a rare association between CD and hemochromatosis, and therefore, more studies in this field are necessary to substantiate this observation.

Pathogenesis of liver dysfunction in CD

**Figure 1:**
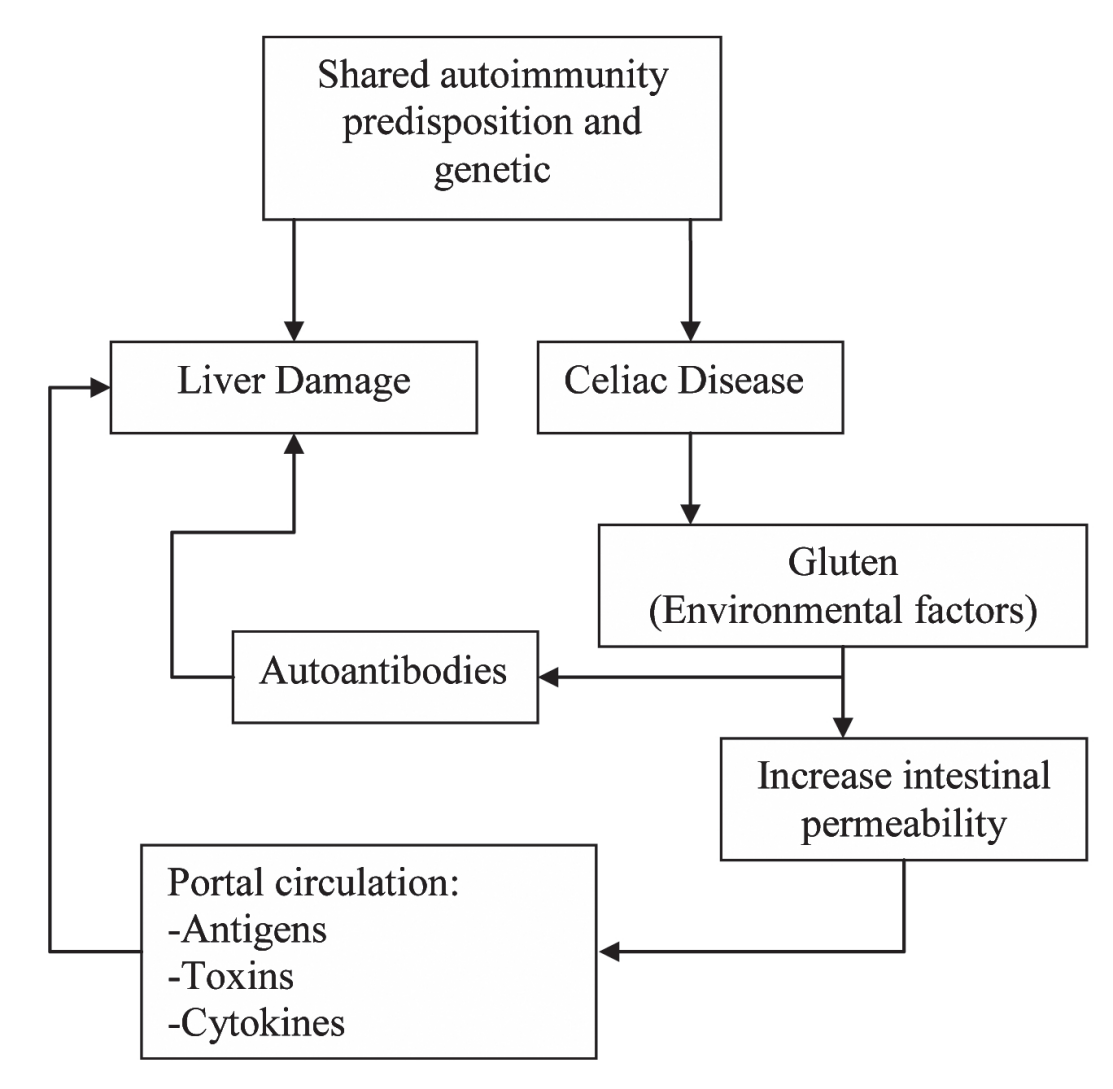
Possible pathogenic mechanisms of liver abnormalities in CD

The mechanism(s) of liver injury in CD is still undefined. Intestinal permeability is increased in CD; this may enable the entry of toxins, antigens, and inflammatory materials to the portal circulation, and these mediators may have a role in the liver involvement seen in patients with CD [14][28][29][56][99]. Although liver dysfunction occurs not only in patients with CD but also in those with irritable bowel disease (IBD), cow milk enteropathy, and food allergies, patients with tropical sprue and increased intestinal permeability do not present with liver enzyme abnormalities as frequently as CD patients [56] (Figure 1). Most patients with liver injury associated with CD have no symptoms or signs of liver disorder at the time of diagnosis, although nonspecific symptoms such as malaise and fatigue are common [14][27][28]. The sensitivity and specificity of each sign is variable, but a combination of signs may otherwise raise the suspicion of CD. However, mild to moderate elevations in serum levels of aspartate aminotransferase (AST) and/or ALT are the most common and often the only laboratory manifestations of liver injury in patients with CD. In a study of 98 confirmed CD patients with varied LFT, there was an increase in only ALT levels in 8 patients and AST levels in 5 of these 8 patients (unpublished data) After adherence to a GFD for 6 months, elevations in serum aminotransferase levels were normalized, thus explaining the correlation between liver injury and intestinal damage.

Moreover, in view of the response to GFDs in hypertransaminasemia patients and abnormal ALP of suspected hepatic origin in CD patients, a liver biopsy may be useful. However, liver biopsy performance is dependent to some extent on factors such as patient age, associated co-morbid conditions, and the clinical significance of the liver test abnormality. Before interaction between environmental and genetic factors lead to irretrievable liver injury, early recognition of CD would allow complete recovery from hepatic lesions. The following mechanisms have been suggested to have roles in establishing liver injury in CD: malabsorption and long-standing malnutrition, increased intestinal permeability, small intestine bacterial overgrowth, chronic intestinal inflammation, and a common genetic predisposition.

Furthermore, patients with CD who are exposed to gluten during a gluten challenge are at higher risk to develop autoimmune disease than those without further exposure [1][103].

Genetic Correlation

Genetic predisposition has an important role in the development of cryptogenic liver disorders in response to autoimmune hepatic injuries, and this might also contribute to the association between CD and liver disease [57]. Different studies reflect the importance of genetic factors in the pathogenesis of CD, which is associated with HLA class II molecules encoded by genes of the HLA complex on chromosome 6 [104]. In this region, a large group of autoimmune diseases are linked to specific alleles or combinations of haplotypes [105]. The main genetic marker of CD is the HLA-DQ2 (α1*0501, α1*0201) heterodimer, which is present in approximately 95% patients with CD, and the remaining patients have HLA-DQ8 (α1*0301, β1*0302) [106]. HLA-DQ2 is in strong linkage with HLA-DR3, which is also the major HLA risk factor for AIH [107]. In addition, AIH is associated with HLA-DR4 and HLA-DR52 [108][109]. The same HLA class II molecule has an important role in the pathogenesis of PSC and CD. A shared immunogenetic predisposition to autoimmunity may account for the association between these diseases [33][110][111]. In a multicenter study in Europe, it was found that the frequencies of the HLA-DR3, HLA-DR13, HLA-DQ2, HLA-DQ8, and HLA-DQB1*0603 haplotypes were higher in patients with PSC compared with matched controls [112]. In Finnish adults who had undergone liver transplantation, the frequency of HLA-DQ2 and HLA-DQ8 in patients with PSC, autoimmune hepatitis, or acute liver failure was 56-75% compared with 39% in controls [33]. Therefore, we suggest that there is a closer association between CD and AIH/PSC than between CD and other liver disorders.

## Conclusion

**Figure 2:**
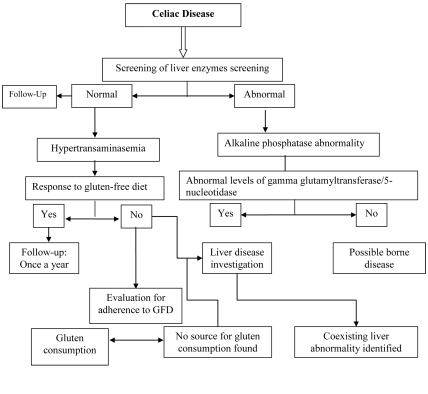
Approach to the diagnosis of liver abnormalities in celiac disease

Liver abnormality may be one of the associated extraintestinal manifestations of CD. However, because of the high frequency of CD in the general population, an accidental association between CD and the liver cannot be excluded. The liver damage in CD ranges from mild hepatic abnormalities to severe liver disease and may be seen in 15-55% of patients. The mechanisms underlying liver abnormalities in CD are not defined clearly. However, consumption of a GFD is an effective treatment for most patients with CD and liver disorders. After excluding other causes of liver disease and because of the high prevalence of liver disorders in CD, the levels of liver enzymes should be evaluated in all patients at the time of CD diagnosis (Figure 2).

In cases of cryptogenic hypertransaminasemia, transaminase levels will be normalized, and it is recommended to re-check the liver enzyme levels after 6-12 months of a strict GFD. Fatty liver also may be associated with CD, although it is unclear whether this is a cause, an effect, or a serendipitous association. The important point is that 10-20% of this group may show hypertransaminasemia again after 2 or more years of GFD because of metabolic changes in the liver owing to the high amount of lipids found in commercially available gluten-free foods. For this reason, monitoring of transaminase levels once a year is recommended, especially in patients gaining weight. Since the association of CD with liver autoimmunity has been largely validated, the first step is requesting a serology test for CD in all cases of autoimmune liver disorders such as AIH, PBC, and PSC. Indeed, CD can be present in 3-7% of these patients.

EMA is more specific but slightly less sensitive than anti-tTG [113], and as is well known, false-positive results for anti-tTG are found in patients with liver and autoimmune disorders [114]. Therefore, determination of the levels of EMA is preferred to that of anti-tTG in patients with cryptogenic and autoimmune liver disease. An exception is in children with inflammatory liver disease of unknown cause, where investigation for CD should be started by determining the anti-tTG levels. In conclusion, serological evaluations for CD should be part of the general workup of patients with unexplained elevated liver enzyme levels when other causes of liver disease have been ruled out, and at the time of CD diagnosis, liver dysfunction should be concurrently evaluated.
